# Different responses to two types of 5-fluorouracil prodrugs in combination with interferon-alpha in pulmonary metastases of renal cell carcinoma: a case report

**DOI:** 10.1186/1757-1626-2-6567

**Published:** 2009-05-26

**Authors:** Tatsuo Morita, Kazuhiko Nakano, Masayuki Yuzawa

**Affiliations:** Department of Urology, Jichi Medical UniversityYakushiji 3311-1, Shimotsuke-city, Tochigi 3290498Japan

## Abstract

A 66-year-old Japanese man with pulmonary metastases of renal cell carcinoma found 8 months after radical nephrectomy was treated with interferon-alpha and tegafur-uracil. Since it failed to achieve tumor responses resulting in progression, he was given interferon-alpha and capecitabine. After 2 courses of combination therapy with IFN-alpha and capecitabine, significant tumor responses were obtained; two out of four pulmonary metastatic sites disappeared completely, one site showed over 50% decrease in size, and the remaining one site did no change in size. The regimen was well tolerated and toxicity observed was World Health Organization grade 1 anorexia. His disease status was maintained as stable disease by the repeated treatment with interferon-alpha and capecitabine for 17 months after tumor responses were obtained. However, tumor progression was observed thereafter. He is at present under treatment with sorafenib. This is the first case report of metastatic renal cell carcinoma, which showed different responses to two types of 5-fluorouracil prodrugs in combination with interferon-alpha, suggesting the biochemical modulation of capecitabine by interferon-alpha as a possible mechanism underlying the antitumor effect of the combination of interferon-alpha and capecitabine at the clinical setting. Present case also suggests that a combination of tumor-selective capecitabine with interferon-alpha is a potentially useful therapeutic option in metastatic renal cell carcinoma.

## Introduction

Current standard therapy against metastatic renal cell carcinoma (RCC) is moving from the cytokine-based therapy to the inhibition of angiogenesis with targeted agents. Since the latter is indeed promising but is not curative, we need to explore the further treatment regimens, which would benefit RCC patients. Cytokines, such as interleukin-2 (IL-2) and/or interferon (IFN) produce responses in 10-15% of patients, with occasional complete responses reported [1]. On the other hand, tumor response rates of 13% to 43% in the treatment of metastatic RCC have been reported by immunochemotherapy consisting of IFN-alpha and fluoropyrimidines with or without IL-2 [2-4]. Capecitabine is an orally administered, tumor-selective fluoropyrimidine that is converted to 5-fluorouracil (5FU) by three enzymes: carboxylesterase mainly located in the liver, cytidine deaminase in the liver and tumors, and thymidine phosphorylase (TP) in tumors [5]. In clinical trials in metastatic RCC, combination of IFN-alpha and tumor-selective capecitabine has a slightly superior overall response rate compared with capecitabine monotherapy: 12-24% [6,7] versus 4.8-8.7%, respectively [8,9]. Herein, we report a case of pulmonary metastases of RCC, which were resistant to the treatment with IFN-alpha and tegafur-uracil (UFT) but were sensitive to the treatment with IFN-alpha and capecitabine.

## Case presentation

**Figure 1.**
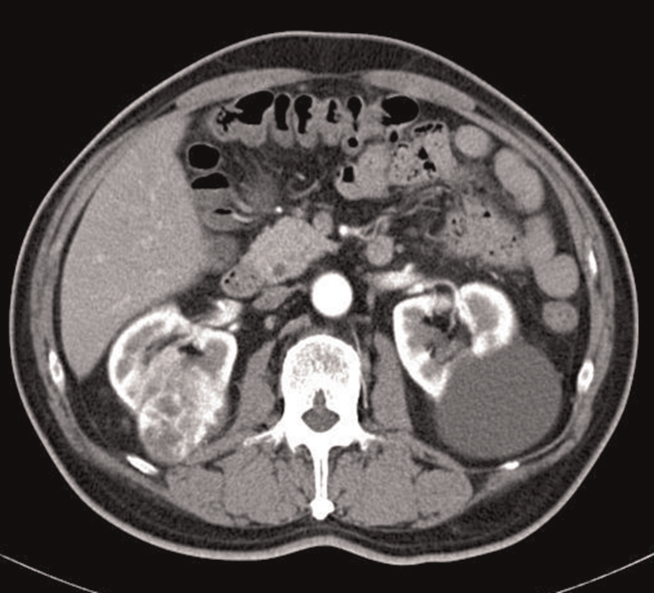
Abdominal CT before radical nephrectomy. Abdominal CT showed right hypervascular renal mass compatible with RCC.

**Figure 2.**
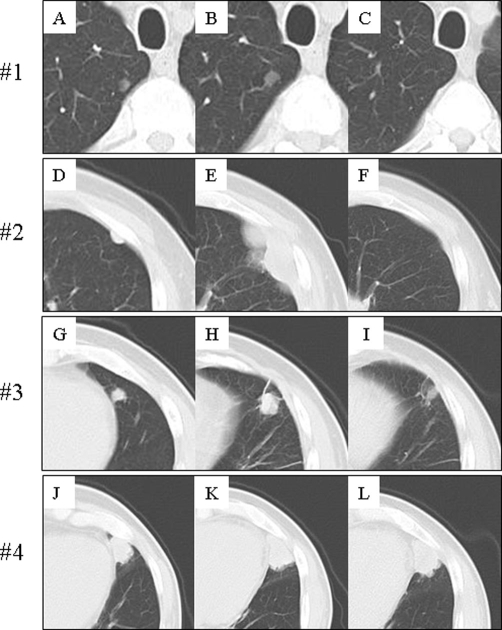
Chest CT before and after the combination therapy with IFN-alpha and capecitabine. Pulmonary metastases were found 8 months after radical nephrectomy (**A**,**D**,**G**,**J**). Combination therapy with IFN-alpha and UFT failed to achieve tumor responses and PD was observed (**B**,**E**,**H**,**K**). Two (#1, #2) out of four pulmonary metastatic sites disappeared completely, one site (#3) showed over 50% decrease in size, and the remaining one site (#4) did no change in size after 2 courses of the combination therapy with IFN-alpha and capecitabine (**C**,**F**,**I**,**L**).

A 66-year-old Japanese man with right RCC (cT2N0M0) which was detected incidentally by ultrasound of health checkup underwent radical nephrectomy on October 2005 (Figure 1). Pathological examination revealed clear cell carcinoma with an alveolar arrangement (pT3N0). After the operation, he was followed up periodically without any further treatments. On June 2006, systemic examination demonstrated four coin lesions in bilateral lungs consistent with multiple pulmonary metastases of RCC without any other metastatic sites as shown in Figure 2 (A, D, G, J). He received combination therapy with IL-2, IFN-alpha (Sumiferon^TM^), and UFT as reported previously [10]. It was discontinued immediately because of the skin toxicity due to IL-2. Then, he was given UFT at a daily dose of 300 mg three times a day and natural IFN-alpha (Sumiferon^TM^) 3 million U intramuscularly five times a week in a course of 3 weeks on/1 week off as the first line treatment. However, chest computed tomography (CT) showed progression of the disease (PD) after two courses of the treatment. We switched 3 million U of IFN-alpha (Sumiferon^TM^) to 5 million U of natural IFN-alpha (OIF^TM^) in combination with UFT based on a case report that metastatic RCC which did not respond to natural IFN-alpha (Sumiferon^TM^) did respond to another type of natural IFN-alpha (OIF^TM^) [11]. However, two courses of natural IFN-alpha (OIF^TM^) in combination with UFT as the second line treatment failed to achieve tumor responses and PD was observed again as shown in Figure 2 (B, E, H, K). Combination therapy with IFN-alpha (OIF^TM^) and UFT was discontinued. On May 2007, he was given capecitabine at a daily dose of 2400 mg twice a day and natural IFN-alpha (OIF^TM^) 5 million U intramuscularly three times a week in a course of 2 weeks on/1 week off as the third line treatment. After 2 courses of the combination therapy with IFN-alpha and capecitabine, significant tumor responses were obtained; two out of four pulmonary metastatic sites disappeared completely, one site showed over 50% decrease in size, and the remaining one site did no change in size as shown in Figure 2 (C, F, I, L). This regimen was well tolerated and toxicity observed was World Health Organization (WHO) grade 1 anorexia without hand-foot syndrome. His disease status was maintained as stable disease by the repeated treatment with IFN-alpha and capecitabine for 17 months after tumor responses were obtained. However, tumor progression was observed thereafter. He is at present under treatment with sorafenib.

## Discussion

Present case is unique in terms of different responses to two types of 5FU prodrugs. Namely, pulmonary metastases of RCC resistant to the treatment with IFN-alpha and UFT were sensitive to the treatment with IFN-alpha and capecitabine. Following possible explanations may account for the different responses to two types of 5FU prodrugs in combination with IFN-alpha Firstly, TP expression in tumor tissues might be involved in the causes of different responses to two types of 5FU prodrugs. TP is a rate-limiting enzyme for capecitabine and involved much in the anabolic pathway of capecitabine rather than UFT. Ishikawa et al. [12] showed that TP expression level in tumor tissues correlated well with the efficacy of capecitabine but not UFT in human cancer xenograft model, suggesting that TP expression level would be a predictive factor for capecitabine. TP expression in RCC is also independent prognostic factor for RCC patients and varies among them [13-15]. Thus, we examined and evaluated TP expression in the resected kidney of the present case by immunohistochemistry as reported previously [14], and confirmed that it had medium to high TP expression level (data not shown). Although TP expression level in primary site is not always the same as that in metastatic sites, immunohistochemistry suggests that RCC in the present case might be sensitive to capecitabine in terms of TP expression resulting in the significant tumor responses to capecitabine. Secondly, biochemical modulation in which a modulator combined with anti-tumor drugs changes pharmacokinetics and results in increased efficacy and/or reduction of toxicity would be expected in capecitabine when administered in combination with IFN-alpha. Transfection studies [16] showed direct evidence that enhancement of TP expression by transfection of TP cDNA makes RCC cell line more susceptible to capecitabine not only in vitro but also in vivo, suggesting that enhancement of TP expression in tumor tissues by modulators such as IFN-alpha would increase capecitabine efficacy. We examined modulatory effect of IFN-alpha on 5FU and 5′-deoxy-5-fluorouridine (5′DFUR) which is the metabolite in the anabolic pathway of capecitabine with a particular focus on TP expression in human RCC cell lines [17], and showed that IFN-alpha up-regulates TP expression and modulates fluoropyrimidine anabolism resulting in enhancement of the sensitivity to 5FU [17] and 5′DFUR [18] in RCC cell lines. Furthermore, extent of enhancement of the sensitivity to 5′DFUR by IFN-alpha was higher than that in 5FU [18]. Similar results were reported using human colon cancer cell line COLO 201 [19]. Taken together, we believe that biochemical modulation of capecitabine by IFN-alpha via TP might contribute to the significant tumor responses in the present case. Thirdly, the dose of UFT used in the present case might be insufficient to achieve the tumor responses. Although optimal dose of UFT in Japan ranges 300 mg to 600 mg, we employed 300 mg UFT because we experienced severe side effect of leukoencephalopathy-like symptoms in metastatic RCC patient in whom complete response (CR) was obtained by the combination of 600 mg UFT and IFN-alpha as reported previously [20]. Akaza et al. reported that combination of 300 or 600 mg UFT and IFN-alpha showed 3 CR cases and 2 partial response (PR) cases in 25 cases with metastatic RCC with a overall response rate of 20% [21]. Therefore, it cannot be excluded that 300 mg UFT used in the present case was insufficient to achieve the tumor responses.

## Conclusion

To our knowledge, this is the first case report of metastatic RCC which showed different responses to two types of 5FU prodrugs in combination with IFN-alpha, suggesting the biochemical modulation of capecitabine by IFN-alpha as a possible mechanism underlying the antitumor effect of the combination of IFN-alpha and capecitabine at the clinical setting. Present case also suggests that a combination of tumor-selective capecitabine with IFN-alpha is a potentially useful therapeutic option in metastatic RCC.

## List of abbreviations

CT: Computed tomography
IL-2: interleukin-2
IFN: Interferon
PD: Progression of the disease
RCC: Renal cell carcinoma
TP: Thymidine phosphorylase
UFT: Tegafur-uracil
WHO: World Health Organization
5′DFUR: 5′-deoxy-5-fluorouridine
5FU: 5-fluorouracil
